# Exploring How People Interact With Dental Misinformation on Social Media: A Cross-Sectional Study

**DOI:** 10.7759/cureus.56625

**Published:** 2024-03-21

**Authors:** Alaa Alhomsi, Hala Aldoss, Abeer A. Aljoujou, Ammar Mahmoud Mashlah, Mohammad Y. Hajeer, Amr Alyafi, Imad Addin Almasri

**Affiliations:** 1 Department of Oral Medicine, Faculty of Dentistry, University of Damascus, Damascus, SYR; 2 Department of Orthodontics, Faculty of Dentistry, University of Damascus, Damascus, SYR; 3 Department of Applied Statistics, Faculty of Economics, University of Damascus, Damascus, SYR

**Keywords:** medical information, misleading information, evidence-based digital policy, facebook, telegram, whatsapp, instagram, dental misinformation, health-related information, social media

## Abstract

Background: Social media has been widely utilized to spread health-related information, which has led to the possibility of spreading medical misinformation. The extent of the issue is unknown, even though policymakers, researchers, and medical professionals agree that health misinformation must be controlled and combated.

Objective: A cross-sectional study was conducted by the faculty of dentistry at Damascus University.

Methods: This quantitative cross-sectional study used a voluntary questionnaire distributed online in social media groups. Participants were of different ages, genders, and educational levels.

Results: Of the 301 questionnaires collected, 291 were analyzed. The study sample comprised secondary education certificates, university students, and postgraduate students. 96.6% of the study sample used two or more SM applications. 71.5% of the study sample uses Facebook more than other applications for dental information research, followed by Instagram (14.8%), Telegram (8.2%), and finally WhatsApp (5.5%). There was a significant difference between the age of the respondents and the SM application they used most (p = 0.027). 46.4% of the study sample were neutral in their answer about what they believed in the trust of information on SM sites, followed by 42.3% who did not agree with the trust of information on SM sites and 11.3 who agreed. 89.7% of the study sample visit the dentist when facing a medical problem, while 10.3% follow advice on SM sites. There was a significant difference between the age of respondents and the source they relied on when facing a medical problem (p = 0.019). 50.2% of the study sample sometimes feels that the advice on SM sites is marketing for a company or product or advertising for doctors and not others.

Conclusion: In particular, understanding the methods and prevalence of health misinformation, as well as the current gaps in knowledge in this area, will help us to guide future research and, more specifically, to develop evidence-based digital policy action plans targeted at addressing this public health issue through various social media platforms.

## Introduction

With one million new users joining the internet daily, there is no indication that the global digital growth trend will slow down. Social media (SM) use is fueled by this increase, as 3.5 billion people, or 45% of the world´s population, currently utilize social media. Social networking sites are online spaces where users can contact one another and exchange private messages, images, videos, and many other types of information [1]. The Internet has greatly aided information exchange, especially in the medical field. Public participation is now essential to creating and generating public health information on the internet rather than being a passive consumer [2]. In the social media-based environment of health information sharing, there is a remarkable deal of potential for misinformation and miscommunication, even though it offers many opportunities for empowerment [3].

False or misleading information that poses as real news is called fake news. Since they are widely shared on social media and the internet, many readers who lack knowledge mistakenly think they are real. They are often invented in jest or to sway political opinion. Misinformation is disseminating an inadvertent incident that can be fixed [4]. At least since the early days of printing, misinformation has been disseminated for a long time. In 1925, an article in Harper´s Magazine titled "Fake News and the Public" lamented how newswires allowed the quick dissemination of false information. The piece also gave rise to the term "fake news," which has gained significant importance today. Social media has helped the public receive scientific data more quickly, but it has also increased the sensationalist language used to explain these findings [5]. The most well-known social media platforms are Facebook, WhatsApp, YouTube, Instagram, Snapchat, Twitter, and more, with steadily growing user bases [6]. Dental disinformation can result in the use of dangerous or unproven therapies, the rejection of reasonable preventative measures, the mistrust of medical professionals, and the propagation of dental myths. Furthermore, SM gives nonexpert influencers and pseudo-experts the ability to spread misinformation and opinions about health care [7].

Therefore, the aim of the study was to focus on the major role that social media plays in people’s access to dental medical information and how it has affected people of different ages, genders, and educational levels. Also, to find out which application people use most to obtain dental and medical information, find out whether people use social networking sites as a reliable source for obtaining dental and medical information, and find out whether social media plays a role as a marketing means for some companies or products or an advertising means for doctors and not others.

## Materials and methods

Study design and setting

This cross-sectional study was conducted at Damascus University's Faculty of Dentistry in 2022/2023. The University of Damascus's Local Research Ethics Committee approved this research project (approval number: 14202206). Furthermore, this investigation was conducted in compliance with applicable guidelines and regulations.

Study instrument

Surveys are a good way to learn about a population's opinions and beliefs. This study was a voluntary questionnaire distributed online in social media groups. Participants were of different ages, genders, and educational levels. A questionnaire was developed based on similar studies related to the topic and written in Arabic. The questions were formatted according to the study objectives while avoiding lengthy questions to ensure a higher response rate with less possibility of missing data.

The questionnaire consists of eight questions, three of which are related to demographics (age, gender, and educational level). Five questions related to the use of SM (the number of applications they use, the site they use most, the degree of their trust in the medical information they obtain from social media sites, the primary source they turn to when they have a medical problem, and their feelings about advice from medical communication sites if they are marketing for a company or product or as an advertising means for a doctor). The questionnaire was first updated by specialists (specialist colleagues in dentistry and statistics).

The questionnaire was pilot tested on 10 people to check their understanding of the questions and express their opinions if they found anything mysterious. Some of the questions were changed to make the questionnaire clearer and easier to comprehend based on the feedback and ideas from the respondents, and then it was published online. The final version was created and made available online using the survey-generating application Google Forms. No registration is necessary, and the service can be accessed through various electronic devices, including tablets, computers, and smartphones. About three minutes should be needed for completion.

Data collection and analysis

The questionnaire was available in various Facebook groups and some WhatsApp groups. To reach a wider range of Syrians, the researchers uploaded a survey link and asked Facebook and WhatsApp users with multiple attributes and demographics to share it. However, Facebook and WhatsApp are becoming increasingly relevant as social networks, so they were selected as the preferred social media for the research survey. Completing the study did not require payment; participation was entirely voluntary.

No identifying information was collected to maintain anonymity. By default, the data would not be collected in Google Forms if the questionnaire had not been completed. If the respondents indicated they were under 18, their responses were not included in the analysis. Participants gave their permission to participate by sending in their filled-out survey forms.

Statistical analysis

Demographic data are presented as descriptive statistics and were carried out to assess the primary features of the study participants. Statistical analysis was conducted through IBM Corp. Released 2017. IBM SPSS Statistics for Windows, Version 25.0. Armonk, NY: IBM Corp. The non-parametric Chi-square independence test was used to evaluate the distribution of measurement variables. A p-value of less than 0.05 was considered statistically significant for all analyses. We also used Microsoft Excel (Redmond, USA) to clarify the results, using appropriate charts and shapes.

## Results

Of the 301 questionnaires collected, 10 were excluded (six declared that they were under 10 years old; two respondents failed to provide informed permission; two questionnaires were identical duplicates before the internet connection was cut off while the respondents were answering). A total of 291 respondents were included in the analysis. The study sample comprised secondary education certificates, university students, and postgraduate students. Their ages ranged from 18-20 years to more than 35 years. Demographical data are shown in Table 1.

**Table 1:** Demographical data of the sample study

Variable		Educational level							
		Secondary education certificates		University students		Postgraduate students		Total	
		frequency	percentage	frequency	percentage	frequency	percentage	frequency	percentage
Gender	female	56	19.2%	169	58.1%	29	10%	254	87.3%
	male	12	4.1%	16	5.5%	9	3.1%	37	12.7%
Total		68	23.4%	185	63.6%	38	13.1%	291	100%
Age	18 to 20 years	16	5.5%	3	1%	0	0%	19	6.5%
	20 to 25 years	28	9.6%	78	26.8%	4	1.4%	110	37.8%
	25 to 35 years	12	4.1%	70	24.1%	30	10.3%	112	38.5%
	Older than 35 years	12	4.1%	34	11.7%	4	1.40%	50	17.2%
Total		68	23.4%	185	63.6%	38	13.1%	291	100%

The sample’s study responses to the question “How many applications do you use?” were 96.6 of them using two or more SM applications, 2.7% using one application, and 0.7% not using any SM applications. There were no significant differences between the genders, ages, and academic degrees of respondents and the number of SM applications they used (p>0.05; Table 2).

**Table 2:** The study sample’s answers to the question “How many applications do you use?” according to their gender, age, and educational levels a: Chi-square test; SM: Social media

Variable			Number of SM applications they used			Total	p-value
			Do not use any SM application	Use one application	Use two or more SM application		
Total			0.7%	2.7%	96.6%	100%	0.863^ a^
gender	female	Frequency	2	7	245	254	
		percentage	100%	87.5%	87.2%	87.3%	
	male	Frequency	0	1	36	37	
		percentage	0%	12.5%	12.8%	12.7%	
Total		Frequency	2	8	281	291	0.835^ a^
		percentage	100%	100%	100%	100%	
age	18 to 20 years	Frequency	0	1	18	19	
		percentage	0%	12.5%	6.4%	6.5%	
	20 to 25 years	Frequency	1	3	106	110	
		percentage	50%	37.5%	37.7%	37.8%	
	25 to 35 years	Frequency	0	3	109	112	
		percentage	0%	37.5%	38.8%	38.5%	
	Older than 35 years	Frequency	1	1	48	50	
		percentage	50%	12.5%	17.1%	17.2%	
Total		Frequency	2	8	281	291	
		percentage	100%	100%	100%	100%	
Educational level	secondary education certificates	Frequency	1	2	65	68	0.699 ^a^
		percentage	50%	25%	23.1%	23.4%	
	university students	Frequency	1	6	178	185	
		percentage	50%	75%	63.3%	63.6%	
	postgraduate students	Frequency	0	0	38	38	
		percentage	0%	0%	13.5%	13.1%	
Total		Frequency	2	8	281	291	
		percentage	100%	100%	100%	100%	

The questionnaire was used to assess the type of SM use. 71.5% of the study sample uses Facebook more than other programs, followed by Instagram at 14.8%, then Telegram at 8.2%, and finally WhatsApp at 5.5%. There was a significant difference between the respondent's age and the SM application he used most (p=0.027; Table 3). 46.4% of the study sample were neutral in their answer about what they believed in the trust of information on SM sites, followed by 42.3% who did not agree with the trust of information on SM sites and 11.3 who agreed. There was no significant difference between the genders, ages, and academic degrees of respondents and their trust in medical information on SM sites (p>0.05).

**Table 3:** The study sample’s answers to the question “What site do you use most to obtain dental and medical information?” are distributed according to their gender, age, and educational levels * significant at the 0.05 level, a: Chi-square test

Variable			The application they used the most					
			Facebook	Instagram	Telegram	WhatsApp	Total	P-value
Total			71.5%	14.8%	8.2%	5.5%	100%	0.72 ^a^
Gender	female	Repetition	179	39	21	15	254	
		percentage	86.1%	90.7%	87.5%	93.8%	87.3%	
	male	Repetition	29	4	3	1	37	
		percentage	13.9%	9.3%	12.5%	6.3%	12.7%	
Total		Repetition	208	43	24	16	291	
		percentage	100%	100%	100%	100%	100%	
Age	18 to 20 years	Repetition	12	5	2	0	19	0.027*^a^
		percentage	5.8%	11.6%	8.3%	0%	6.5%	
	20 to 25 years	Repetition	71	19	15	5	110	
		percentage	34.1%	44.2%	62.5%	31.3%	37.8%	
	25 to 35 years	Repetition	83	17	6	6	112	
		percentage	39.9%	39.5%	25%	37.5%	38.5%	
	Older than 35 years	Repetition	42	2	1	5	50	
		percentage	20.2%	4.7%	4.2%	31.3%	17.2%	
Total		Repetition	208	43	24	16	291	
		percentage	100%	100%	100%	100%	100%	
Educational level	secondary education certificates	Repetition	53	8	7	0	68	0.32^ a^
		percentage	25.5%	18.6%	29.2%	0%	23.4%	
	university students	Repetition	127	30	14	14	185	
		percentage	61.1%	69.8%	58.3%	87.5%	63.6%	
	postgraduate students	Repetition	28	5	3	2	38	
		percentage	13.5%	11.6%	12.5%	12.5%	13.1%	
Total		Repetition	208	43	24	16	291	
		percentage	100%	100%	100%	100%	100%	

89.7% of the study sample visit the dentist when facing a medical problem, while 10.3% follow the advice on SM sites. There was a significant difference between the age of respondents and the source they relied on when facing a medical problem (p=0.019; Table 4). 39.8% of those who visit the dentist are between the ages of 25 and 35, while 60% of those who rely on the advice on SM sites are between the ages of 20 and 25.

**Table 4:** The study sample’s answers to the question “The main source I rely on when I face a medical problem” according to their gender, age, and educational level * significant at the 0.05 level, a: Chi-square test; SM: Social media

Variable			The main resource rely on when they have a medical problem			P-value
			Visit the dentist	Rely on the advice on SM sites	Total	
Total			89.7%	10.3%	100%	0.493^ a^
Gender	female	Frequency	229	25	254	
		percentage	87.7%	83.3%	87.3%	
	male	Frequency	32	5	37	
		percentage	12.3%	16.7%	12.7%	
Total		Frequency	261	30	291	
		percentage	100%	100%	100%	
Age	18 to 20 years	Frequency	16	3	19	0.019*^a^
		percentage	6.1%	10%	6.5%	
	20 to 25 years	Frequency	92	18	110	
		percentage	35.2%	60%	37.8%	
	25 to 35 years	Frequency	104	8	112	
		percentage	39.8%	26.7%	38.5%	
	Older than 35 years	Frequency	49	1	50	
		percentage	18.8%	3.3%	17.2%	
Total		Frequency	261	30	291	
		percentage	100%	100%	100%	
Educational level	secondary education certificates	Frequency	57	11	68	0.19^ a^
		percentage	21.8%	36.7%	23.4%	
	university students	Frequency	169	16	185	
		percentage	64.8%	53.3%	63.6%	
	postgraduate students	Frequency	35	3	38	
		percentage	13.4%	10%	13.1%	
Total		Frequency	261	30	291	
		percentage	100%	100%	100%	

50.2% of the study sample sometimes feel that the advice found on SM sites is marketing for a company or product or an advertising means for doctors and not others, followed by 44.7% of the study sample who often find this advice is marketing for a company or product or an advertising means for doctors and not others, followed by 5.2% of the study sample who do not find it as marketing advice (Table 5).

**Table 5:** The study sample’s answers to the question: “Did you feel that some of the advice on social media sites is marketing for a company or product or specific doctors and not others?” according to their gender, age, and educational level. a: Chi-square test

Variable			I feel that some of the advice on social media sites is marketing for a company or product or specific doctors and not other				p-value
			often	sometimes	never	Total	
Total			44.7%	50.2%	5.2%	100%	0.726^ a^
Gender	female	Frequency	114	126	14	254	
		percentage	87.7%	86.3%	93.3%	87.3%	
	male	Frequency	16	20	1	37	
		percentage	12.3%	13.7%	6.7%	12.7%	
Total		Frequency	130	146	15	291	
		percentage	100%	100%	100%	100%	
Age	18 to 20 years	Frequency	5	14	0	19	0.118^ a^
		percentage	3.8%	9.6%	0%	6.5%	
	20 to 25 years	Frequency	43	58	9	110	
		percentage	33.1%	39.7%	60%	37.8%	
	25 to 35 years	Frequency	58	50	4	112	
		percentage	44.6%	34.2%	26.7%	38.5%	
	Older than 35 years	Frequency	24	24	2	50	
		percentage	18.5%	16.4%	13.3%	17.2%	
Total		Frequency	130	146	15	291	
		percentage	100%	100%	100%	100%	
Educational level	secondary education certificates	Frequency	23	42	3	68	0.184^ a^
		percentage	17.7%	28.8%	20%	23.4%	
	university students	Frequency	86	88	11	185	
		percentage	66.2%	60.3%	73.3%	63.6%	
	postgraduate students	Frequency	21	16	1	38	
		percentage	16.2%	11%	6.7%	13.1%	
Total		Frequency	130	146	15	291	
		percentage	100%	100%	100%	100%	

## Discussion

Internet users have increasingly used social media over the past 20 years to exchange and look for health information [8]. Regardless of gender or age, users of health information from all social groups participate more frequently on these social media platforms [9]. While these platforms have been beneficial for promoting health, new research indicates that misinformation about health may spread more quickly through social media than accurate scientific information. Thus, it is critical to understand how health disinformation spreads and how it may influence choices and health-related behaviors [10].

The term "health misinformation" is becoming more common in our society, but because of the wide range of health themes and the natural dynamics of the social media ecosystem, its definition is becoming more and more elusive. We here describe health misinformation as a health-related assertion based on anecdotal evidence that is inaccurate or misleading due to the absence of existing scientific knowledge, using a broad term that can incorporate the vast diversity of meanings in the scientific literature [5]. Since they are widely shared on social media and the internet, many readers who lack knowledge mistakenly think they are real. They are often invented in jest or to sway political opinion. Misinformation is disseminating an inadvertent incident that can be fixed [4]. The self-perceptions and independent actions of digital users from a hyperconnected age drive the rising creation and consumption of inaccurate or misleading online health information [5].

The huge amount of false material found on social media that directly impedes the prevention and management of chronic diseases has raised issues in recent studies. The results of the previous research show that over 20% of misleading information originates from a single source and that 40% of fake news is disseminated on social media [11]. Very few studies were conducted to evaluate dentists' awareness of disinformation and fact-checking. Fake news articles on dentistry have been more prevalent in recent years; these include articles promoting homemade whitening products and incorrect dental advice. With 93.1% of our respondents using SM daily and a third accessing SM more than 10 times daily, our respondents were heavy SM users. This is also documented in the literature, raising the possibility that using SM could become addictive, particularly given that today's students view the internet as an extension of who they are [12]. In addition, several new social media platforms-like Instagram Reels, TikTok, and Snapchat-have become prominent [13]. 75.1% of our study sample uses Facebook more than other applications. Additionally, Facebook is the most popular social media network worldwide and the most visited dental information website. Instagram is gaining popularity quickly, especially among teenagers and young people.

Rajeh et al. study, evaluating the use of social media to obtain medical information for students of three different universities in the western region of the Kingdom of Saudi Arabia, found that 97.5% used WhatsApp the most, while Facebook was the least used program, 25.1% [14]. In addition, in the study by Arnett et al., Facebook was the most used program at a rate of 57.3%. We can attribute these percentage differences to the different societies in which the three studies were conducted [15]. 11.3% of our study sample agreed that information on social networking sites is reliable. However, Scherer et al.'s study found that 80% of users believe that information found online is authentic, which supports the idea that people share deceptive stuff on social media more frequently than reliable content [16]. The use of charcoal toothpaste and other healthy products to strengthen teeth is among the latest dental health "fake news" that has gained popularity. Because they are incorrectly thought to be "toxin," the latter things are commonly advised without fluoride. The ongoing advertising of these items on various social media platforms enhances their perceived authority. Prior research has indicated that individuals searching the Internet for information on oral health typically have a keen interest in dental caries, fluoride, molar incisor hypomineralization, and toothaches. Given the detrimental effects of health myths and the paucity of dentistry research on information disorder [17]. Due to the difficulty in understanding messages caused by the overload of content seen in modern information ecosystems, people may adopt unfavorable health beliefs that impair their ability to make decisions and manage specific problems on their own, which can harm their quality of life [18]. Therefore, the dentistry community should hope that the highly accessible and educationally valuable material available to patients is of excellent quality [19]. A subtler but no less dangerous kind of false information has recently crept into the dental community. On social media and healthcare websites nowadays, it is common to find online advertisements for dental products or services that purport to be scientific or professional advice.

The literature mentions that dental professionals were mostly responsible for creating and spreading false information, typically connected to alternative therapies and driven primarily by financial interests [20]. US medical schools realized 10 years ago that there were no policies regarding the use of SM, but since then, they have created SM guidelines. Dental practitioners and clinics use SM to publish and upload images and videos of their patients as part of marketing campaigns (also known as "before and after shots"), instructional resources, and professional self-promotion. Research indicates that regulatory organizations like medical boards have the authority to take disciplinary action in response to blatant transgressions of professional behavior, such as lying about qualifications, inappropriate patient communication, and confidentiality [21].

Professionals need to properly market their offerings to avoid exacerbating the problem of disinformation. Dentists should never stop providing trustworthy, factually correct online dental health materials. To improve patients' oral health literacy, dentists and organized dentistry must teach people how to assess the validity of online health information. Patients need to learn from dentists how to evaluate the reliability and relevance of oral health information, any possible bias, the credentials of the authors, and the availability of scientific proof. The following elements need to be taken into account when confirming the facts: (1) verify the source of the information, (2) look past the news stories, (3) check the supporting references, (4) look at the responses of other readers if they agree, (5) double-check if the information is a joke, (6) consider the possibility of prejudices, (7) speak with experts, and (8) reconsider sharing before doing it.

Limitations of the current study

This study has several limitations. One constraint pertains to the approach employed in gathering pertinent data via an electronic questionnaire. Many of the intended audience either don’t use these kinds of communication networks or don’t reply to electronic surveys. The absence of a probability sampling method is the second potential limitation. This could impact how well the results apply to the entire Syrian population. Therefore, the generalizability of the current research findings is limited.

## Conclusions

Several strategies exist to mitigate false information: Sources supporting the user's beliefs should be made available; false information propagated by bots and "cyborg" accounts should be recognized; and algorithms should be tuned to overlook these tricks. Users share less information overall because they are cautioned that it can be inaccurate.

In particular, understanding the methods and prevalence of health misinformation, as well as the current gaps in knowledge in this area, will help us to guide future research and, more specifically, to develop evidence-based digital policy action plans targeted at addressing this public health issue through various social media platforms.
